# *Trypanosoma cruzi* Experimental Infection Impacts on the Thymic Regulatory T Cell Compartment

**DOI:** 10.1371/journal.pntd.0004285

**Published:** 2016-01-08

**Authors:** Florencia Belén González, Flavia Calmon-Hamaty, Synara Nô Seara Cordeiro, Rodrigo Fernández Bussy, Silvana Virginia Spinelli, Luciano D'Attilio, Oscar Bottasso, Wilson Savino, Vinícius Cotta-de-Almeida, Silvina Raquel Villar, Ana Rosa Pérez

**Affiliations:** 1 Institute of Clinical and Experimental Immunology of Rosario (IDICER CONICET-UNR), Rosario, Argentina; 2 Laboratory on Thymus Research, Oswaldo Cruz Institute, Oswaldo Cruz Foundation, Rio de Janeiro, Brazil; 3 Laboratory of Innovations in Therapy, Teaching and Bioproducts, Oswaldo Cruz Institute, Oswaldo Cruz Foundation, Rio de Janeiro, Brazil; Universidade Federal de Minas Gerais, BRAZIL

## Abstract

The dynamics of regulatory T cells in the course of *Trypanosoma cruzi* infection is still debated. We previously demonstrated that acute murine *T*. *cruzi* infection results in an impaired peripheral CD4^+^Foxp3^+^ T cell differentiation due to the acquisition of an abnormal Th1-like phenotype and altered functional features, negatively impacting on the course of infection. Moreover, *T*. *cruzi* infection induces an intense thymic atrophy. As known, the thymus is the primary lymphoid organ in which thymic-derived regulatory T cells, known as tTregs, differentiate. Considering the lack of available data about the effect of *T*. *cruzi* infection upon tTregs, we examined tTreg dynamics during the course of disease. We confirmed that *T*. *cruzi* infection induces a marked loss of tTreg cell number associated to cell precursor exhaustion, partially avoided by glucocorticoid ablation- and IL-2 survival factor depletion. At the same time, tTregs accumulate within the CD4 single-positive compartment, exhibiting an increased Ki-67/Annexin V ratio compared to controls. Moreover, tTregs enhance after the infection the expression of signature markers (CD25, CD62L and GITR) and they also display alterations in the expression of migration-associated molecules (α chains of VLAs and chemokine receptors) such as functional fibronectin-driven migratory disturbance. Taken together, we provide data demonstrating profound alterations in tTreg compartment during acute murine *T*. *cruzi* infection, denoting that their homeostasis is significantly affected. The evident loss of tTreg cell number may compromise the composition of tTreg peripheral pool, and such sustained alteration over time may be partially related to the immune dysregulation observed in the chronic phase of the disease.

## Author Summary

Regulatory T cells (Tregs) play a key role in balancing protective immunity and pathogenesis during diverse parasitic infections. In the context of *Trypanosoma cruzi* infection, some findings showed that peripheral Tregs (pTregs) might have an important role as contraregulatory mechanism, limiting tissue damage and avoiding the more severe clinical forms of chronic Chagas disease. Furthermore, there are finding showing that murine lethal *T*. *cruzi* infection causes a severe depletion of pTregs and the induction of T-bet and IFN-γ expression in the remaining pTregs, which might favours immunopathology. The thymus is a central organ of the immune system that sustains thymic Tregs (tTregs) development, but which is also a target of *T*. *cruzi* infection. We previously showed profound alterations of CD4^+^CD8^+^ double positive and CD4^−^CD8^−^double negative populations during infection. The present study confirms that *T*. *cruzi* also severely influences the thymic compartment of tTreg cells, resulting in a series of phenotypic, locational and functional disturbances. These findings showed that a parasitic infection could alter the normal homeostasis of tTregs, while in the context of *T*. *cruzi* infection these data indicate that tTregs disturbances may influence the development of chronic pathology, considering the suspected autoimmune basis of chagasic cardiomyopathy.

## Introduction

Regulatory T cells (Tregs) herein defined as CD4^+^Foxp3^+^ T cells represent a population that plays an essential role in the maintenance of self-tolerance and in the shutdown of inflammatory response. According to their origin, two major classes of Tregs have been described: thymus-derived Tregs (tTregs) and peripherally-derived Tregs (pTregs). The tTreg population is differentiated in the thymus and populates the periphery early, around day 3 of life; whereas in periphery, environmental antigens or other signals can up-regulate Foxp3 in conventional CD4^+^T cells, converting them into pTregs [1]. Tregs are also characterized by the expression of certain surface markers, mainly the IL-2 receptor α chain or CD25, which is expressed constitutively in this population. IL-2, together with other cytokines from the same family like IL-15, favours Tregs expansion, maturation and survival [2–4].

In the context of infections, Tregs play a special role in controlling the magnitude of immune activation and are also involved in the restoration of the homeostatic environment [5]. However, the studies of Treg dynamics in infectious settings have been mainly focused on the involvement of pTreg population, and little is still known the potential impact of infections upon the thymic compartment of Tregs. Given that tTreg homeostasis is likely to be altered during infectious processes, abnormalities at thymic level may have harmful consequences for host immunocompetence.

Chagas disease (also known as American trypanosomiasis) is caused by the protozoan parasite *Trypanosoma cruzi* (*T*. *cruzi*) and represents one of the most frequent endemic parasitic diseases in Latin America. Nowadays, Chagas disease has acquired global relevance because is spreading to non-endemic countries. The disease has a wide spectrum of symptoms and outcome, ranging from an asymptomatic infection, to an acute illness or a chronic gastrointestinal or cardiac disease.

The role played by Tregs during *T*. *cruzi* infection is still controversial and diverse hypotheses have been proposed. An unfavorable role of Tregs during the infection seems plausible either because a defective or excessive function in the partially autoimmune basis for chronic chagasic cardiomyopathy, or parasite persistence, respectively [6–8]. In this regard, it is noteworthy that studies conducted in both humans or experimental settings have evaluated Treg dynamics in blood, secondary lymphoid organs or heart [8–12], but there is no available information regarding the potential impact of *T*. *cruzi* infection on the tTreg population.

It is well established that severe *T*. *cruzi* murine infection induces a strong Th-1 response accompanied by a marked thymic atrophy [13]. In this respect, we previously demonstrated that atrophy is mainly caused by massive apoptosis of cortical CD4^+^CD8^+^ double positive (DP) thymocytes induced by raised glucocorticoid levels [13–15]. Interestingly, other non-mutually exclusive mechanisms appear to be involved in the evolution of this atrophy, such a decrease in cell proliferation, and an increase in the thymic output of mature and immature thymocytes [16], while is suspected a low income of T-cell progenitors from the bone marrow [16]. Moreover, a variety of alterations in the migration pattern of thymocytes were observed in association with the anomalous expression of receptors and ligands for extracellular matrix proteins, cytokines or chemokines [17–19]. Furthermore, our recent results clearly show important phenotypic and functional changes in the pool of pTregs during infection [12], suggesting that tTregs are also affected.

Herein, we investigated whether experimental acute *T*. *cruzi* infection impacts upon the dynamics of tTregs. Our results provide a clear demonstration that *T*. *cruzi* infection caused profound abnormalities in the tTreg compartment within the thymus.

## Materials and Methods

### Mice and experimental infection

C57BL/6 and BALB/c male mice, aged 6–8 weeks were obtained from the animal facilities at Rosario Medical School and Oswaldo Cruz Foundation. Trypomastigotes of the Tulahuen or Y strain of *T*. *cruzi*, corresponding to *T*. *cruzi* lineage VI and II respectively [20], were used. Mice were infected subcutaneously with 100 or 1,000 viable trypomastigotes. To monitor the systemic repercussion of the acute disease, parasitemia and the survival time was recorded following infection.

### Ethics statement

Experiments with mice were performed in strict accordance with the recommendations in the Guide for Care and Use of Laboratory Animals of the National Institute of Health and were approved by each Institutional Ethical Committee (School of Medical Sciences from National University of Rosario, Bioethics and Biosecurity Committees, Resolution N° 3740/2009, and Oswaldo Cruz Foundation Ethics Committee on Animal Use, Resolution P-0145-02).

### Flow cytometry analysis

Thymuses were removed, minced and thymocytes were washed and incubated in PBS containing 3% fetal calf serum (Gibco, California, USA). For immunostaining, 1x10^6^ thymocytes were incubated with a given mix of specific fluorochrome-conjugate monoclonal antibodies for 30 min at 4°C in the dark: PerCP/anti-CD4, APC/anti-CD4, FITC/anti-CD8, PECy7/anti-CD8, FITC/anti-CD62L, FITC/anti-GITR, PerCP/anti-CD4 (from BD Pharmingen, San Diego, USA), FITC/anti-CD4, PE/anti-Foxp3, APC/anti-CD25, PE/anti-CCR7/CD197 (from eBioscience), FITC/anti-VLA-4α chain/CD49d, PE/anti-VLA-6α chain/CD49f, PE/anti-CXCR4/CD184 (from BD Pharmingen, Franklin Lakes, USA) and FITC/anti-VLA-5α chain/CD49e (Southern Biotechnologies, Birmingham, USA). For intracellular staining, cells were incubated in fixation/permeabilization buffer (eBioscience, Mouse Regulatory T cell Staining kit, USA) for 1 h, then resuspended on permeabilization buffer (eBioscience, USA) and incubated for 30 min with the specific monoclonal antibody, following the manufacturer’s guidelines. Once defined the lymphocyte gate, events were collected in a FACSAria II Flow cytometer (BD Biosciences, USA). Data were analyzed using DiVa (BD Biosciences) or FlowJo (Tree Star, USA) softwares. Negative control stainings were defined from fluorochrome-conjugated antibody isotype controls.

### Determination of cell death and proliferation

Assessment of thymocyte death by annexin V labeling (BD Pharmingen) was carried out according to the manufacturer’s instructions. Briefly, thymocytes were washed in annexin V binding buffer, and 1x10^6^ cells were stained with FITC-labeled annexin V, followed by permeabilization and PE-Foxp3 staining, as described previously [12]. In all cases, flow cytometry was performed immediately after staining. Dead cells were gated on the basis of forward and side scatter parameters. For proliferation studies, FITC- or PE-coupled antibodies against Ki-67 were added together with anti-Foxp3 antibody and subjected to fixation/permeabilization procedures, as described above. For each sample, at least 100,000 events were collected in a FACSAria II Flow cytometer. Data were analyzed using DiVa or FlowJo softwares.

### Adrenalectomy

Mice were anesthetized with 100mg/kg ketamine and 2mg/kg xylazine and afterward bilateral adrenalectomy was performed via dorsal approach, as previously published [21]. Briefly, two small incisions were made on each side of the mouse back, just below the rib cage, and the adrenal glands were removed with curved forceps. Sham operation involved similar procedures, but without removing the adrenals. Following the operation, adrenalectomized mice were supplemented with 0.9% (wt/vol) sodium chloride in drinking water. Animals were infected one week after the surgery.

### Cell migration assay

For evaluation of the *in vitro* thymocyte migratory response towards fibronectin, of 5-μm-pore transwell inserts (Corning Costar, Cambridge, USA) were coated with 10 mg/mL of human fibronectin (Sigma Aldrich, USA) for 45 min at 37°C, and non-specific binding sites were blocked with PBS-diluted 1% BSA. Thymocytes were obtained at day 17 post-infection or from non-infected counterparts. Cells (2.5x10^6^ thymocytes in 100 mL of RPMI / 1% BSA) were then added in the upper chambers. Migration medium was serum free, so that to avoid serum-derived fibronectin or other soluble chemotactic stimuli. After 12 hours of incubation at 37°C in a 5% CO_2_ humidified atmosphere, migration rate was defined by counting the cells that migrated to the lower chambers containing migration medium alone (RPMI /1% BSA). Migrating cells were ultimately counted, stained with appropriate antibodies and analyzed by flow cytometry. The percentage of each subset was used along with the total cell counting to calculate absolute numbers of each lymphocyte subset. The results are represented in terms of input seen in each subpopulation, using the following formula, as previously reported [17]:

$$\text{Input }\left(\text{\%}\right)=\frac{Absolute\;number\;of\;migrating\;cells\;with\;a\;given\;phenotype}{Total\;number\;of\;starting\;cells\;with\;a\;given\;phenotype}\;\times 100$$

### Immunofluorescence studies

Thymuses were removed at different days post-infection (p.i.), embedded in Tissue-Tek (Miles Inc., Elkhart, USA), frozen in dry ice and stored at -80°C. Cryostat sections of 3–4 mm-thick were settled on glass slides, acetone fixed and blocked with PBS-diluted BSA 1%. Specific antibodies used were: PE/anti-Foxp3 (eBioscience) and FITC/anti-IL-2 (eBioscience) monoclonal antibodies; anti-cytokeratin (Dako, Glostrup, Denmark), anti-laminin and anti-fibronectin (Novotech, Pyrmont, Australia) polyclonal antibodies; and, as secondary antibodies, anti-goat Alexa 488 and anti-rabbit Alexa 546 (Molecular Probes, Eugene, USA). Sections were incubated with the appropriate antibody for 1 h at 4°C, washed, and in the case of staining for cytokeratin, laminin and fibronectin following subjected to a secondary antibody labeling. Background staining values were obtained subtracting the primary antibody. Samples were analyzed by confocal microscopy using a Nikon Eclipse TE-2000-E2 device (Germany) and the images obtained were subsequently analyzed using the Image J software (Bethesda, Maryland, USA). Immunofluorescence labeling and quantitative confocal microscopy were used to investigate the distribution and/or quantification of Foxp3 and IL-2. Optimal confocal settings (aperture, gain, and laser power) were determined at the beginning of each imaging session and then held constant during the analysis of all samples. The number of cells expressing Foxp3 was evaluated as Foxp3^+^cells/mm^2^. IL-2 fluorescence intensity was measured as an average of each area and the values were recorded as arbitrary units (pixel/μm^2^).

### RNA isolation, cDNA synthesis and qPCR

Thymi were obtained at different days p.i. Total RNA was isolated from cells using TRIzol (Invitrogen, USA) according to the manufacture’s recommendations. mRNA levels were determined by RT-qPCR. cDNA was synthesized from 1 μg of total RNA using Superscript III reverse transcriptase (Invitrogen, USA) and specific primers for murine IL-2 and IL-15. PCR reactions were performed in a StepOne/Plus Real-Time PCR System (Life Technologies, USA) using SYBR Green I (Roche) to monitor dsDNA synthesis. Data were normalized using GAPDH cDNA quantification. Primers sequences were:

IL-2 = F 5’-CCTGAGCAGGATGGAGAATTACA-3'

IL-2 = R 5’-TCCAGAACATGCCGCAGAG-3'

IL-15 = F 5'-AAA CCC ATG TCA GCA GAT AA-3'

IL-15 = R 5'-AAG TAG CAC GAG ATG GAT GT-3'

GADPH = F 5’-AGCAATGCATCCTGCACCACCA-3’

GADPH = R 5’-ATGCCAGTGAGCTTCCCGTTCA-3’

### Statistical analyses

Depending on the characteristics of the variable, differences in quantitative measurements were assessed by ANOVA followed by Bonferroni test or by the Kruskall-Wallis followed by post-hoc comparisons when applicable. Results were expressed as mean ± standard error of the mean (s.e.m) unless otherwise indicated. The Graph-Pad Instat 5.0 software (Graph-Pad, California, USA) was applied for statistical analyses, and differences were considered significant when p value was ≤0.05.

## Results

### *T*. *cruzi* infection induces changes in the frequency and number of tTregs cells

**Fig 1:**
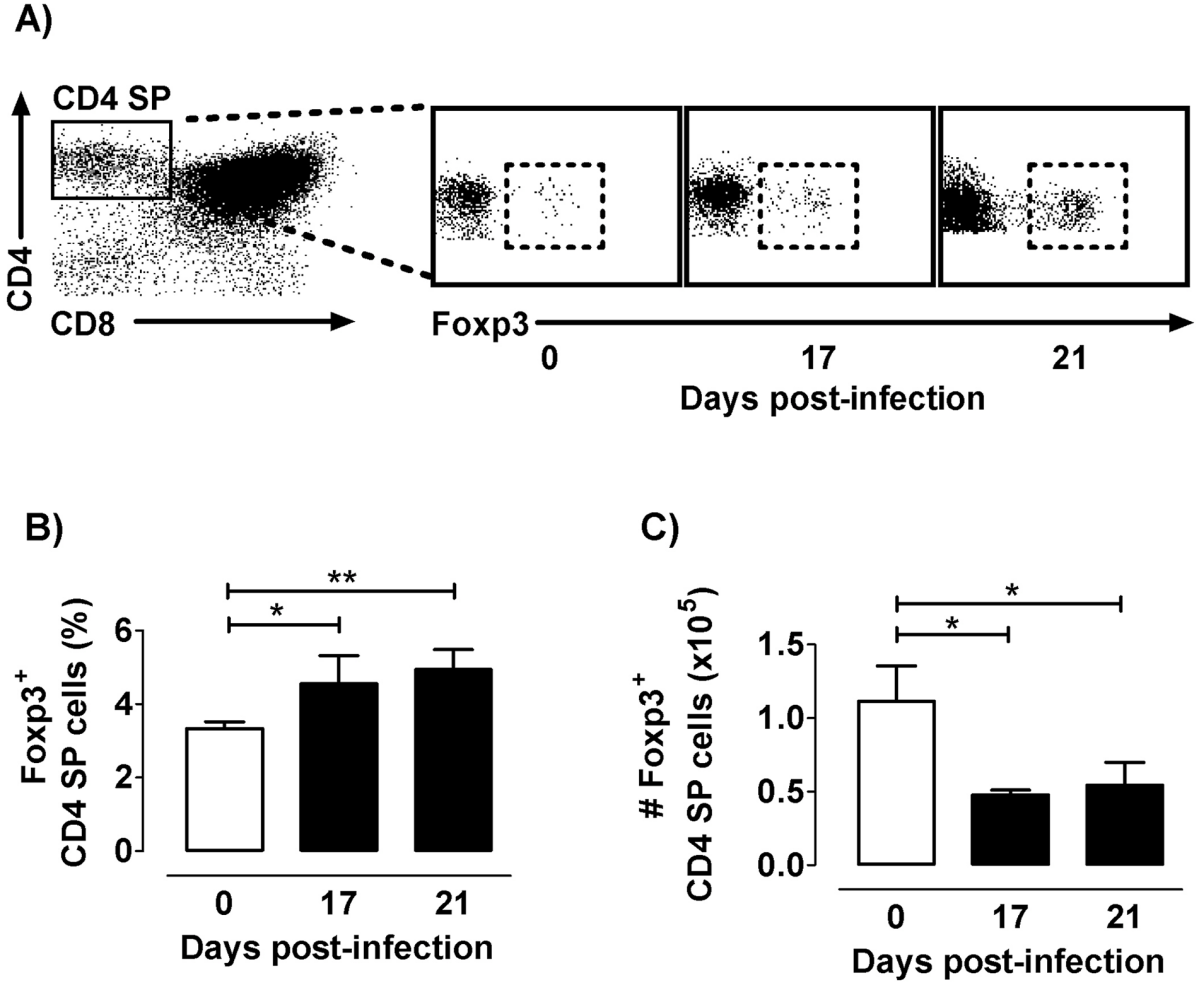
Foxp3 expression among CD4 SP cells along infection. a) Representative dot plots obtained by flow cytometry showing CD4 SP gathered among total thymocytes and Foxp3 expressing cells among CD4 SP cells during the course of infection. b) Frequency of Foxp3^+^ cells among CD4SP thymocytes. c) Absolute number Foxp3^+^ cells among CD4SP thymocytes. Values are mean ± s.e.m. of 5–8 mice/day one representative of six experiments performed independently in C57BL/6 mice infected with Tulahuén strain. * p <0.05 and ** p<0.01.

First, to evaluate whether thymic content of tTregs (detected as Foxp3^+^ cells inside CD4^+^ single-positive -CD4 SP- compartment) were affected, we monitored by cytofluorometry their frequency and cell number during infection in C57BL/6 mice. A progressively increased tTreg frequency was observed within the overall CD4 SP population, raising the highest values by day 21p.i. (Fig 1A and 1B). Nevertheless, there was a dramatic decrease in their absolute numbers (Fig 1C). To evaluate whether these findings were mouse- or parasite-strain dependent, we carried out similar studies in parallel well-established murine models for *T*. *cruzi* infection. Thus, BALB/c and C57BL/6 mice were both infected with either the Tulahuen or Y strains of *T*. *cruzi* [13,22,23]. As shown in S1 Fig, we observed similar results. It follows that *T*. *cruzi* acute infection induces an increase in the proportion of Foxp3^+^cells within the CD4 SP compartment, while reducing their absolute number independently of the mouse or parasite genetic background. In addition, chronically infected mice tended to restore thymic architecture and tTregs numbers (S2 Fig). These findings suggest that specific mechanisms, such as cell death or altered proliferation, may be influencing the homeostasis of Foxp3^+^CD4^+^ SP and also Foxp3^−^CD4^+^ SP thymocytes during the acute phase of infection.

### *T*. *cruzi* infection induces changes in tTreg death and proliferation

**Fig 2:**
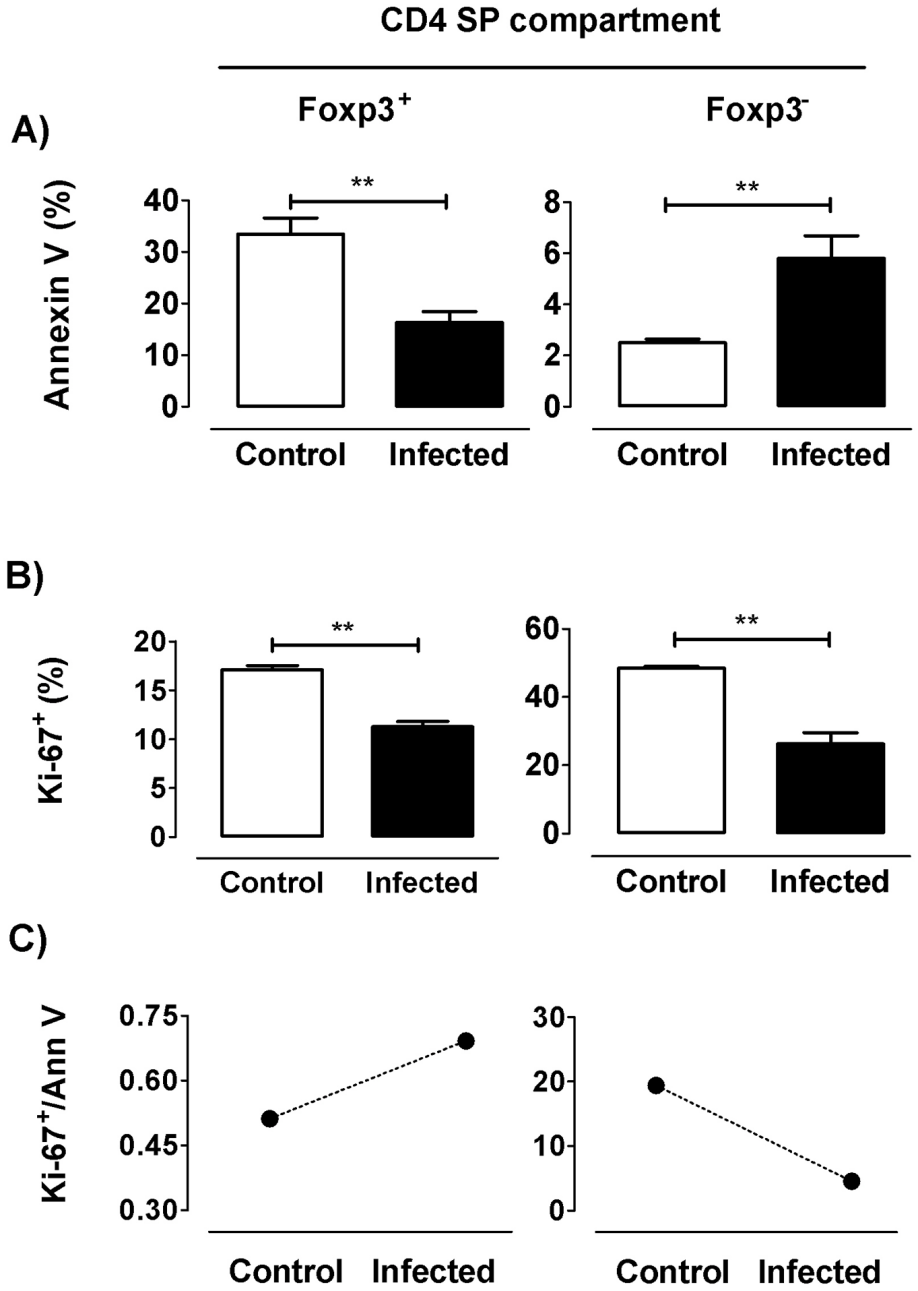
Death and proliferative response of tTregs during *T*. *cruzi* infection. Flow cytometric analysis was achieved in thymocytes from control and 17 days-infected animals. a) Frequency of annexin V^+^ cells among each Foxp3-defined CD4 SP subpopulation. b) Frequency of Ki-67^+^ cells among each Foxp3-defined CD4 SP subpopulation. c) Ki-67/annexin V ratio in control and infected animals within the different subpopulations. Values are mean ± s.e.m. of 3–6 mice/day. One representative of two experiments performed independently in C57BL/6 mice infected with Tulahuén strain. ** p<0.01.

As previously reported, the thymic atrophy is mainly the consequence of an extensive apoptosis of DP cells, although CD4 SP thymocytes are also affected. Thus, the increase in Foxp3^+^ cell proportion within the CD4 SP compartment might be due to a diminished frequency of Foxp3^+^ cell death compared to the corresponding Foxp3^−^ thymocytes. To test this hypothesis, C57BL/6 mice challenged with the Tulahuen strain of *T*. *cruzi* were sacrificed at day 17 p.i. for an ex *vivo* staining of thymocytes with annexin V. The CD4 SP compartment showed an enhanced proportion of cell death (CD4 SP annexin V^+^ cells (%) = day 0: 3±0.7 *versus* day 17 p.i.: 5.7±2.1; p<0.05). Within the CD4 SP population, and beyond the increased basal death levels of Foxp3^+^ compared to Foxp3^−^ cells, the latter population showed a significant increase in annexin V staining after infection; this not being the case among Foxp3^+^ cells in which such staining was visibly diminished (Fig 2A). These results suggest that the enlargement of Foxp3^+^ cell proportions within the CD4 SP compartment during infection may be related to the induction of survival signals in the remaining Tregs, whereas conventional Foxp3^−^ CD4 SP thymocytes became more susceptible to death. An alternative but not excluding explanation for thymic relative accumulation of tTregs within CD4 SP compartment is that Foxp3^+^ cells proliferate more than their Foxp3^−^ counterparts. To check this, we evaluated in both populations the expression of the nuclear antigen Ki-67, a marker cycling cells, as an estimation of proliferation. Strikingly, Foxp3^−^ and Foxp3^+^ cell proliferations were diminished after infection (Fig 2B). Nevertheless, only Foxp3^+^ cells showed an enhanced Ki-67/Annexin V ratio following infection (Fig 2C). Hence, the relative accumulation of tTregs within the CD4 SP compartment may be explained by a better balance of Foxp3^+^ cell cycling *versus* cell death, as compared to Foxp3^−^ cells.

### Decreased numbers of tTregs are partially caused by diminution in DP cell precursors

**Fig 3:**
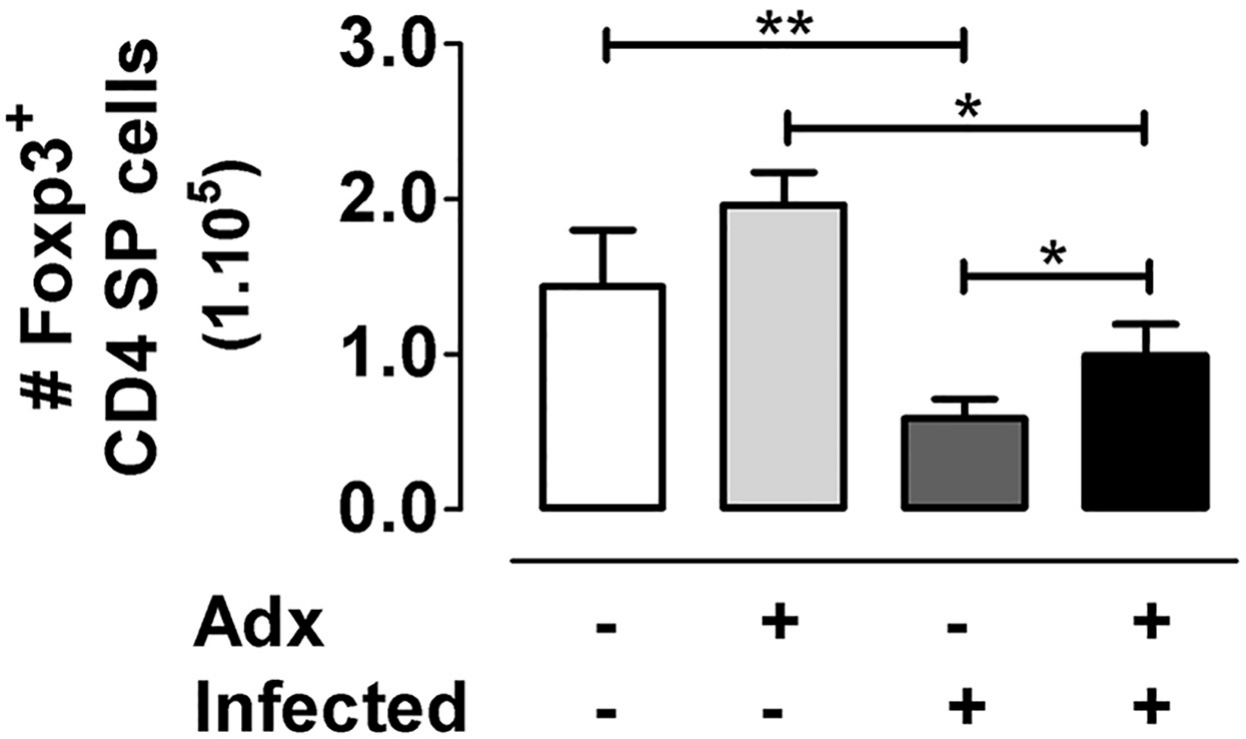
Glucocorticoid depletion and tTreg number during *T*. *cruzi* infection. Animals were subjected to adrenalectomy (Adx) or sham surgery one week after infection. After 14 days p.i., thymuses were obtained and the absolute number of CD4^+^Foxp3^+^ cells was evaluated by flow cytometry. As observed, there was a clear increase in the number of tTregs in Adx+Infected animals compared to the infected counterparts. Results are representative from two independent experimental rounds carried out in C57BL/6 mice infected with Tulahuén strain. Values are mean ± s.e.m. of 4–6 mice/group* p <0.05 and **p<0.01.

Despite the enhanced proportion of Foxp3^+^ cells among CD4 SP thymocytes, tTreg numbers progressively fell in the course of infection, as shown previously in Fig 1. The intrathymic loss of tTregs may be also linked to the depletion of DP cells, which are the most important tTreg cell precursors, at least in numbers. We previously showed that Adx prevented the loss of DP cells induced by glucocorticoids during *T*. *cruzi* infection, although at the same time shortened mouse survival [21]. To test tTreg dynamics in the absence of DP loss driven by glucocorticoids, we performed a bilateral Adx one week after infection. Thymuses were obtained after 14 days p.i. (survival -in days-: Infected = 24.4±1.5; Adx+Infected = 16±2.0) and the absolute numbers of tTregs were evaluated. Although the premature death of Adx+Infected animals is a technical obstacle to carry out studies after 17 or 21 days p.i., the Foxp3^+^ cell reduction after 14 days p.i. was partially prevented by Adx, suggesting that other mechanisms are also operating in the loss of tTregs (Fig 3).

### Decreased absolute numbers of tTregs are linked to a diminution in thymic IL-2 contents

**Fig 4:**
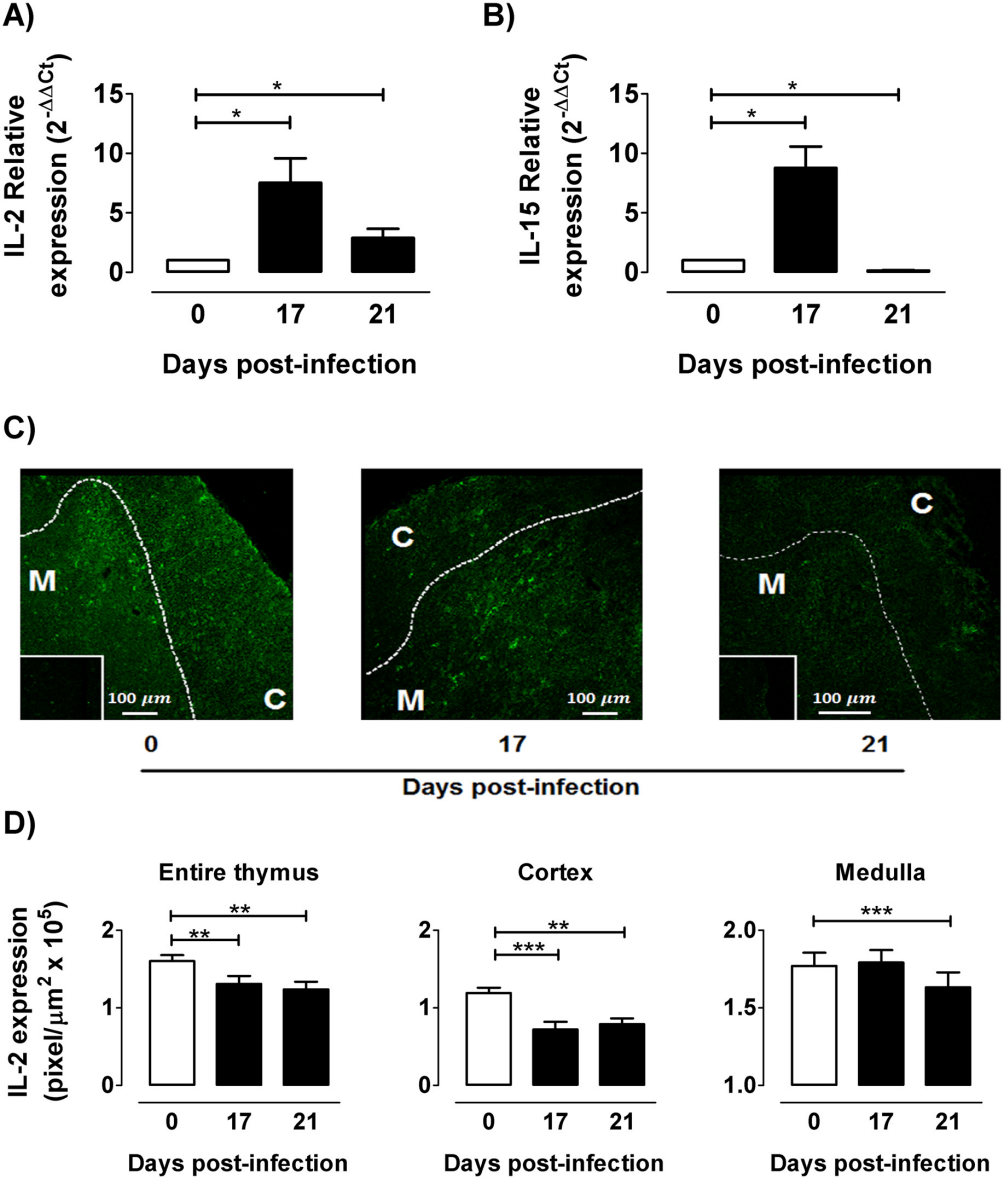
Thymic expression of IL-2 during *T*. *cruzi* infection. After 0, 17 and 21 days p.i., mRNA was extracted from thymuses and quantitative real time RT-PCR was performed for: a) IL-2 b) the IL-2 related cytokine IL-15. In both cases, bars show the relative expression of each thymic transcript using GADPH as reference. Representative results of two independent experiments (n = 3-7/day). For IL-2 localization and semi-quantification, immunofluorescence assays were performed in thymuses from control, 17- and 21 days-infected mice. c) Representative images showed a marked IL-2 inmunoreactivity (green) in both thymic *cortex (C)* and *medulla (M)*, while cortico-medullary boundary is shown by dotted line. Inserts show negative controls of immunostaining. d) IL-2 intensity (pixel/μm^2^) was diminished in thymi from infected animals, both in cortex and medulla. Values are mean ± s.e.m. of 3–6 mice/day. One representative of three experiments performed independently in C57BL/6 mice infected with Tulahuén strain. * p <0.05; ** p<0.01 and *** p<0.001.

The numerical loss of tTregs during infection may be also due to a lack of survival factors, such as IL-2. To test IL-2mRNA and protein levels in the thymus during infection, real time PCR and immunofluorescence experiments were carried out. Expression profiles of IL-2 mRNA showed an increase at day 17 p.i., followed by an evident collapse at day 21 p.i (Fig 4A). The same was true when analyzing mRNA for IL-15 (Fig 4B), a related IL-2 cytokine that also is involved in the transmission signalling through common pathways. In the thymus of non-infected mice IL-2 immunoreactivity was evident in the entire organ; whereas in the infected individuals, IL-2 immunoreactivity progressively diminished (Fig 4C). In addition, in the thymus from 21 days-infected animals IL-2 protein expression was diminished in both medulla and cortex, as seen by fluorescence quantification (Fig 4D). Interestingly, the enhancement of IL-2 mRNA at day 17 p.i. did not correlate with the diminished thymic contents of this cytokine, suggesting that IL-2 transcripts are regulated post-transcriptionally.

### *T*. *cruzi* infection induces enhancement of CD25, GITR and CD62L expression intTregs

**Fig 5:**
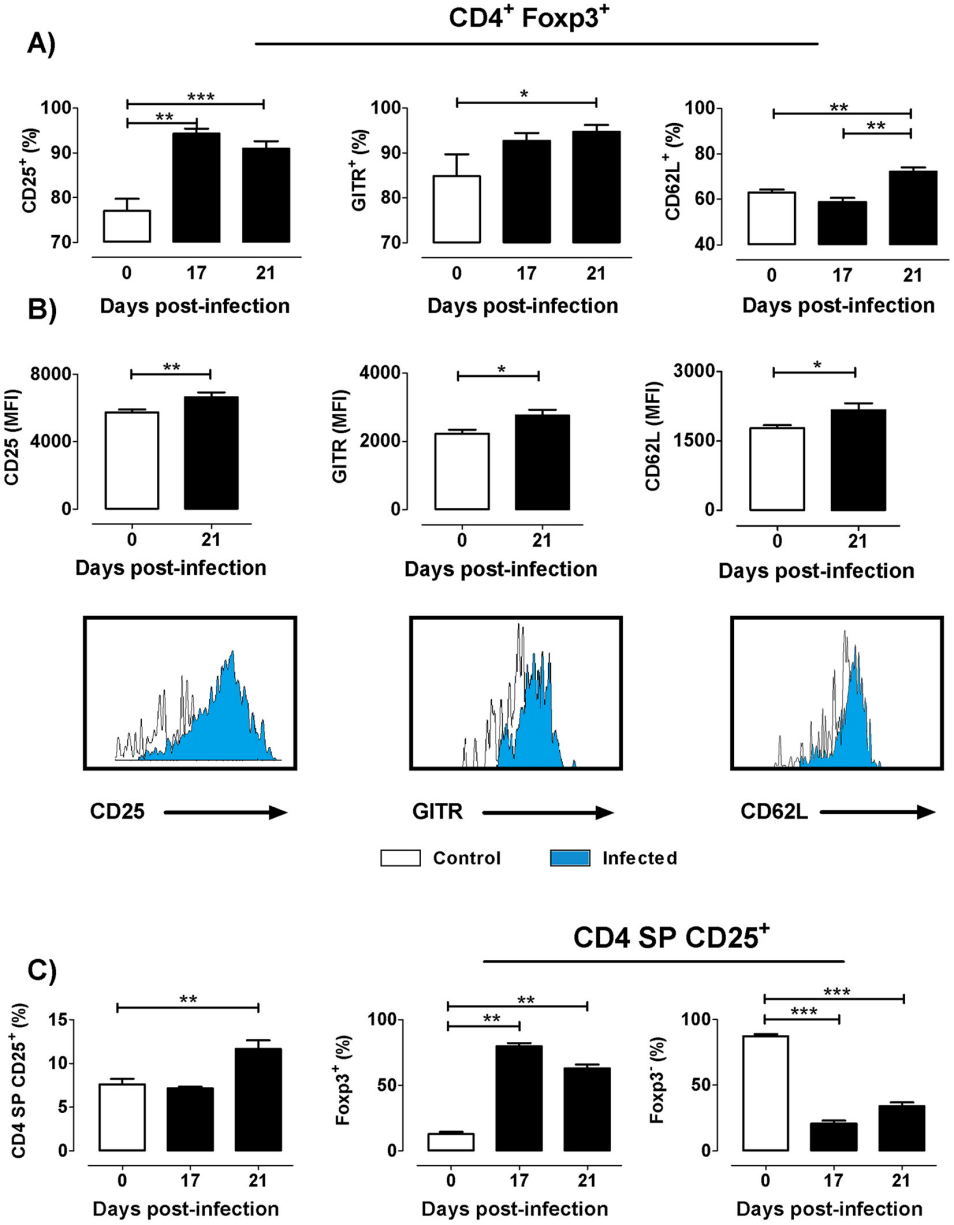
Expression of CD25, GITR and CD62L among Foxp3 expressing cells during *T*. *cruzi* infection. a) Frequency of CD25, GITR, and CD62L expression among CD4^+^Foxp3^+^ cells during the course of infection. b) Mean Fluorescence intensity (MFI) of CD25, GITR and CD62L in CD4^+^Foxp3^+^ and the corresponding representative histograms showing the increase in the MFI of three markers after 21 days p.i. compared to the controls. c) Frequency of CD4^+^CD25^+^ cells within theCD4 SP compartment and Foxp3^+^ and Foxp3^−^ cells within the CD4^+^CD25^+^ population. Values are mean ± s.e.m. of 3–8 mice/day. One representative of three experiments performed independently in C57BL/6 mice infected with Tulahuén strain. * p <0.05; ** p<0.01 and *** p<0.001.

To further evaluate tTregs during experimental *T*. *cruzi* infection, we analyzed the expression of signature molecules, such as CD25, GITR and CD62L. The expression of CD25 and CD62L increased along tTreg maturation. Foxp3^+^ cells expressing CD25 increased ~15% at days 17 and 21 p.i.; with a~10% augment in cells expressing GITR and CD62L being found by day 21p.i. (Fig 5A). An enhancement in the co-expression of CD25^+^GITR^+^ or CD25^+^CD62L^+^ was also observed after infection among CD4^+^Foxp3^+^ cells (S3 Fig). Additionally, the mean fluorescence intensity of the three surface markers was significantly augmented among Foxp3^+^ cells after infection, as shown in Fig 5B Simultaneously, the proportion of Foxp3^−^ cells within the CD4^+^CD25^+^ compartment decreased, suggesting a loss of the most closely tTreg precursors (CD4^+^CD25^+^Foxp3^−^ cells) during infection (Fig 5C). These results indicate that *T*. *cruzi* infection induces a loss of tTreg cell precursors, while the remaining Foxp3^+^ cells display a more differentiated phenotype.

### *T*. *cruzi* infection induces changes in the localization of both cortical and medullary Foxp3 expressing cells

**Fig 6:**
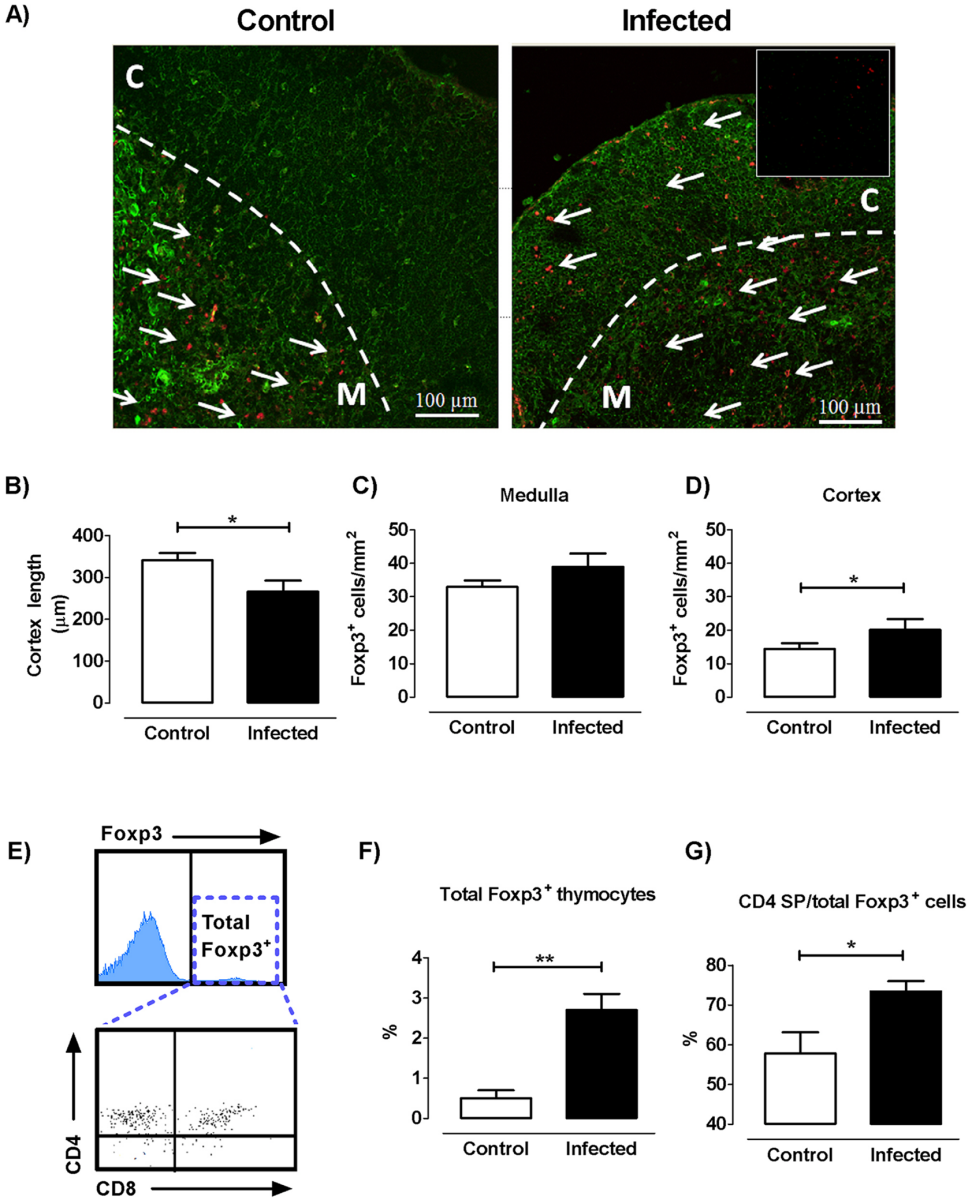
Cortical and medullary location of Foxp3^+^ expressing cells. a) Representative confocal images of thymic lobules from control and infected mice, showing a clear Foxp3^+^ immunostaining (in red fluorescence) in both cortex (C) and medulla (M). Cytokeratins are detected by green fluorescence, while cortico-medullary boundaries are delimited by a dotted line. b) Variations in cortical thickness after 17 days post-infection. c) Number of Foxp3^+^ cells per unit area in the medulla. d) Number of Foxp3^+^ cells per unit area in the cortex. Bars represent the mean ± s.e.m. of data obtained from 10 microscopic fields of thymuses from five control mice and six 17 days-infected mice. Data are representative of two experiments performed independently in C57BL/6 mice infected with Tulahuén strain of parasite. e) Representative analysis of whole thymocytes expressing Foxp3 (Total Foxp3^+^) where dot plots represent Foxp3^+^ expressing thymocytes within the different subpopulations. f) Bars represent the variation in the proportions of whole thymic Foxp3^+^ expressing cells in control and infected thymus. g) Frequency of CD4 SP withinFoxp3 expressing cells. Data correspond to mean ± s.e.m. of 5 mice/group and represent values obtained after 21 days p.i. (one representative of four independent sets of experiments). * p<0.05 and ** p<0.01.

We next carried out double-labeling immunofluorescence studies using pan-cytokeratin along with Foxp3 staining, to analyze the location of Foxp3 expressing cells within the thymic epithelial meshwork. This approach enables an adequate interpretation of Foxp3^+^ cells location in cortical *versus* medullary regions, mainly in infected mouse-derived thymuses given the shrinkage in their cortical area secondary to the DP cell loss. Analyses performed in normal thymuses revealed regions with high immunoreactivity for Foxp3 in the medulla and in a much lesser amount in the cortex (Fig 6A, left panel). The localization of Foxp3^+^ cells in the thymus of infected mice was less restricted to the medulla, since cortical areas with well-defined immunoreactivity began to be detected from day 14 onwards. (Fig 6A, right panel). After 17 days p.i., the shrinkage of cortex reflected the massive loss of DP cells (Fig 6B). In the thymus of control mice, Foxp3^+^ cells in the medulla were on average 2.3 times more frequent than in the cortex (32 vs 14 Foxp3^+^/cells mm^2^, Fig 6C and 6D respectively), whereas in the thymus of 17-day infected mice, this relation was ~ 1.9 (39 vs 20 Foxp3^+^/cells mm^2^, Fig 6C and 6D respectively). Next and for comparative purposes, we selected by cytometry all thymocytes expressing Foxp3 (gate “total Foxp3”), for evaluating Foxp3 expression among thymic subpopulations, as described in Fig 6E. Total Foxp3 expressing thymocytes showed a six-fold increase in the infected thymus compared to controls, after 21 days p.i. (Fig 6F). As expected from earlier results, Foxp3^+^ proportion inside the CD4 SP compartment cells increased significantly at day 21 p.i. (Fig 6G). Unlike to what was expected by microscopy, the fraction and the number of Foxp3^+^ cells inside the DP compartment from infected animals are declined compared to control ones (S4 Fig). In addition, only minor changes were observed in Foxp3^+^ within the CD8 compartment cells after infection (S4 Fig). These results suggest that Foxp3^+^ cortical cells were not necessarily DP cells. It follows that, besides morphological changes noticed in *T*. *cruzi* infected thymus, there is a clearly ectopic location of Foxp3^+^ cells outside the medulla.

### *T*. *cruzi* infection induces variations in the migratory response of tTreg cells

**Fig 7:**
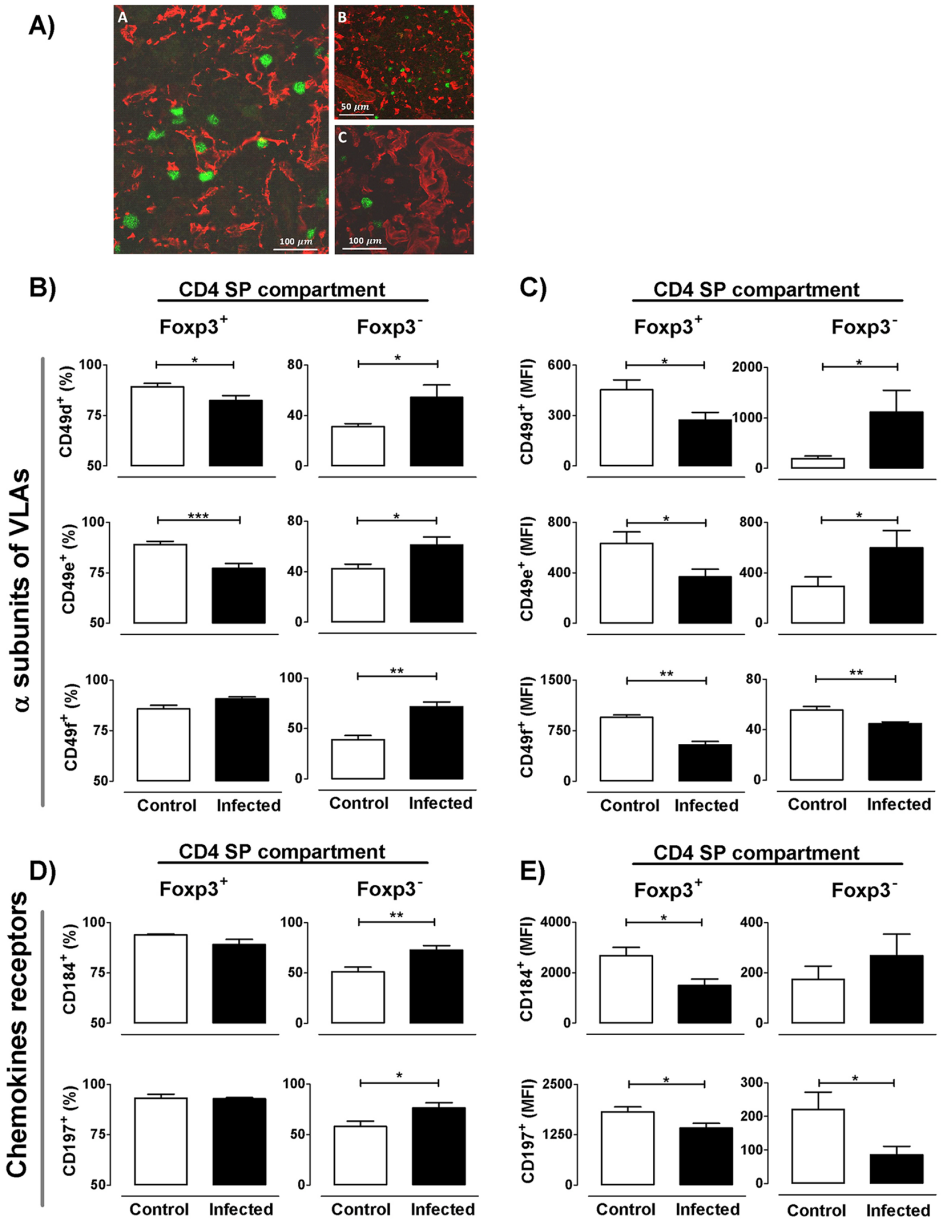
Alterations in migratory-related molecules among Foxp3 expressing cells during infection. a) Localization by immunofluorescence of cytokeratins, extracellular matrix molecules and Foxp3^+^ cells in the thymus of normal (Panel A) and 17 days-infected mice (Panels B and C). Panel A shows medullary Foxp3^+^ cells in close contact with the medullary epithelial stroma (denoted by cytokeratin red staining). Panels B and C show Foxp3^+^ cells located in regions with high extracellular matrix network density, as seen by staining with the anti-laminin antibody (red staining, panel B) and anti-fibronectin (red staining, panel C). b) Frequency of integrin α chains CD49d, CD49e and CD49f within CD4^+^Foxp3^+^ and CD4^+^Foxp3^-^ cells. c) Mean fluorescence intensity (MFI) of each integrin α chain within CD4^+^Foxp3^+^ and CD4^+^Foxp3^-^ cells. d) Frequency of chemokine receptors CD184/CXCR4 and CD197/CCR7 within CD4^+^Foxp3^+^ and CD4^+^Foxp3^-^ cells. e) MFI of each chemokine receptor within CD4^+^Foxp3^+^ and CD4^+^Foxp3^-^ cells. Data are expressed as mean ± s.e.m. of 3–6 mice/day, after 17 days p.i. and exemplify one representative of three experiments performed independently in BALB/c mice infected with Y strain. * p <0.05; ** p<0.01 and *** p<0.001.

Partly because Foxp3^+^ cell frequency was increased inside the CD4 SP compartment of infected animals, and that Foxp3^+^ cells were found in the thymic cortex, we hypothesized that tTregs might have an altered migratory behavior, known to be controlled by distinct signals triggered by integrins and chemokine receptors. Since the thymocyte migratory capacity is linked to their possibility to bind components of the extracellular matrix through receptors belonging to the very late antigen (VLA) family of integrins [19], we first evaluated their expression in both Foxp3^+^CD4 SP and Foxp3^−^CD4 SP cells. In both normal and infected thymuses, Foxp3^+^ cells were located in close contact with the epithelial microenvironmental cells and with the extracellular matrix network, as seen in Fig 7A. Additionally, a decreased proportion of CD49d and CD49e (α chains of fibronectin receptors VLA-4 and VLA-5 respectively) in Foxp3^+^ cells was observed by flow cytometry after infection, while the frequency of CD49f (the α chain of the laminin receptor VLA-6) did not differ (Fig 7B). Moreover, all integrin α chains strongly decreased their mean fluorescence intensity (MFI) after infection (Fig 7C). Conversely, the Foxp3^−^ CD4 SP thymocyte subset showed an increased frequency of cells expressing CD49d, CD49e and CD49f (Fig 7B), accompanied by increased cellular expression levels (as defined by MFI values), except for CD49f (Fig 7C).

We also evaluated in tTregs the expression of two typical tTreg chemokine receptors: CD197/CCR7 and CD184/CXCR4. Notoriously, in normal thymus, the Foxp3^+^ population expressed a higher proportion of both receptors compared to Foxp3^−^ cells (~ 94 vs 51% on average, respectively) (Fig 7C). Moreover, while infection induced an enhancement in their frequency within the Foxp3^−^ population, no differences were found within the Foxp3^+^ subset, which was maintained around 90% (Fig 7C). However, the membrane level of both receptors (ascertained by MFI) was diminished in Foxp3^+^ cells (Fig 7D). Taken together, these findings support the notion that tTreg cells may undergo alterations in their trafficking capacity during *T*. *cruzi* infection.

**Fig 8:**
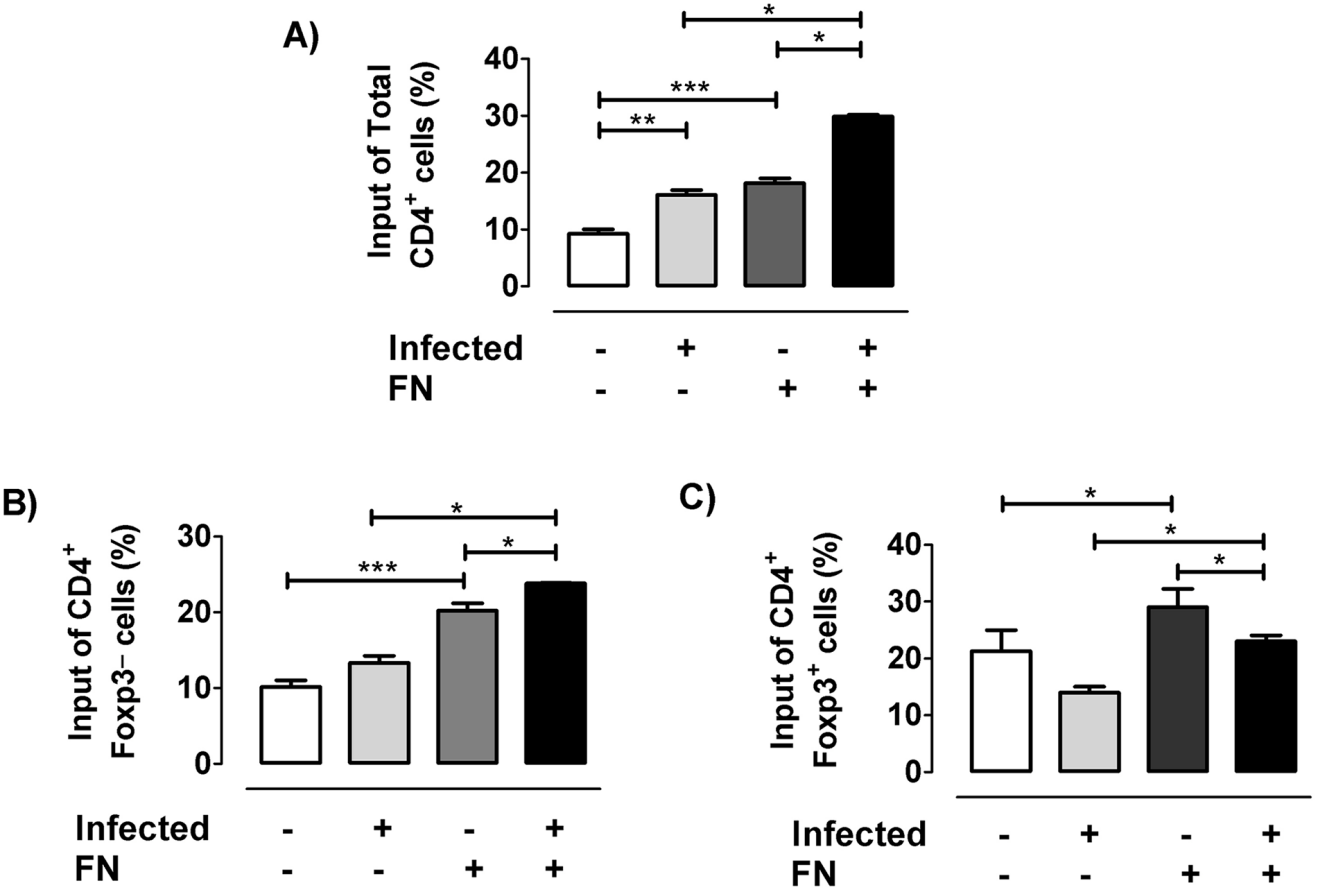
Fibronectin-driven ex vivo migratory activity of tTregs. For migration assays, thymocyte pools of at least 3–4 animals from control or 17 days-infected mice were allowed to migrate in transwell chambers coated with fibronectin (FN+) alone or BSA as control (FN-) during 12 h. a) Percentage of input of total CD4^+^ thymocytes. b) Percentage of input of CD4^+^Foxp3^-^ cells. c) Percentage of input of CD4^+^Foxp3^+^ cells. Flow cytometric analyses of FN-induced migratory activity correspond to the migratory capacity of thymocyte subpopulations as percentage of input (% of input = [Absolute number of migrating cells with a determinate phenotype /Total number of migrating cells] x 100). Results derived from *ex vivo* migration assays are representative from two independent experiments carried out with C57BL/6 mice infected with Tulahuén strain of parasite. *p<0.05 and **p<0,01.

The findings presented here, together our previous reports showing migratory alterations in fibronectin-driven migration of SP or DP subpopulations after infection, led us to hypothesize that the disease can also modify the fibronectin-mediated migratory response of tTreg cells. Using transwell assays for evaluating the *ex vivo* ability of Foxp3^+^ cells to transmigrate, we confirmed that the whole CD4 SP population from infected mice exhibited increased migratory responses through fibronectin-coated surfaces (Fig 8A). Strikingly however, within the CD4 compartment, Foxp3^+^ cells showed the opposite trend. In fact while Foxp3^−^ cells from uninfected animals exhibited an enhanced migration through fibronectin similarly to whole CD4 SP population (Fig 8B), while the fibronectin-driven migration of Foxp3^+^ thymocytes from infected animals was clearly diminished (Fig 8C), thus revealing that *T*. *cruzi* infection induces functional changes regarding fibronectin-driven migratory behaviour of tTregs compared to other thymocyte subpopulations.

## Discussion

Thymic atrophy and DP cell loss during *T*. *cruzi* experimental infection is well documented [13,24–26]. However, no data were available on the potential effect that *T*. *cruzi* infection have on the compartment of tTregs. Here we show that *T*. *cruzi* infection induces a marked loss of tTreg cells, while at the same time this cell population exhibited locational, phenotypic and functional changes.

In healthy animals, the analysis of Foxp3 frequency among different thymocyte subpopulations confirmed that Foxp3^+^ cells roughly represent 3% of CD4 SP cells, coinciding with data previously reported by Liston et al [27]. The same analysis carried out in the thymus of *T*. *cruzi* infected animals showed enrichment on the frequency of Foxp3 expressing cells within the CD4 SP compartment due to an imbalance between cell death and proliferation.

By contrast, there is a patent exhaustion of Foxp3^+^ cell numbers, clearly associated to a DP and CD4^+^CD25^+^Foxp3^−^ cell precursor depletion. Although we previously showed that DP cells die during infection by glucocorticoid-driven apoptosis [28], we cannot discard that other factors account for DP death. These results are in apparent contradiction with data showed by Sanoja *et al*, where the authors observed augmented proportion and numbers of tTregs in mice infected with the Y strain of *T*. *cruzi* [29] This difference may be partly due, at least, to differences in the methodology used in both studies (gender of mice, parasite doses, inoculation route and cytometric analysis).

Medullary CD4^+^CD25^+^Foxp3^−^cells appear to be highly dependent on IL-2 and IL-15 signaling to differentiate into tTregs. Moreover, the appearance of CD4^+^CD25^+^Foxp3^+^ in the medulla depends on IL-2 and IL-15 secretion by CD4 SP cells located in close contact with medullary epithelial cells [30]. Both IL-2 and IL-15 trigger signal pathways on cell precursors through receptor complexes containing the common cytokine γc-chain subunit, which is involved in the activation of Stat5 proteins and Foxp3 expression [2,31,32]. Consequently, the failure of medullary CD4^+^CD25^+^Foxp3^−^ cells to differentiate into tTreg in *T*. *cruzi* infected animals may be the result of reduced thymic contents of IL-2 and IL-15. Accordingly, we propose that the *T*. *cruzi* induce the depletion of cytokines that trigger γc-chain family of receptors or may alter the expression of these receptors, impairing the normal tTreg development [33,34]. Interestingly, during Th-1 polarized responses induced by parasites such as *T*. *cruzi* or *Toxoplasma gondii* there is also a limitation of systemic IL-2 availability [12,35]. Therefore, IL-2 withdrawal favours the collapse of regulatory response and the development of pathology, while the treatment of mice with IL-2 plus glucocorticoids or an IL-2-anti-IL-2 complex can improve Treg response following parasite infection [12,35]. Overall, such findings point out to a relevant role of IL-2 upon thymic and peripheral regulatory response during *T*. *cruzi* infection.

Under physiologic conditions, only a minor proportion of cells differentiate into tTregs. The overrepresentation of Foxp3^+^ cells within the CD4 SP compartment seen during infection, conjointly with a diminished cell death, reveal that this population is more resistant to apoptosis compared to the Foxp3^−^CD4 thymocytes, but not enough to prevent the tTreg loss in absolute numbers. Reinforcing this view, there is an increase in CD25, CD62L and GITR expression among the remaining tTregs. The constitutive expression of CD25 is related to the ability of Foxp3^+^ cells to respond to IL-2, and their expression level has been strongly linked to their survival, cell number, thymic maturational stages and suppressive function [31]. However, in normal animals there is a proportion of Foxp3^+^CD4 SP cells that does not express CD25, and that reaches around 20%; similar to values previously described in thymus and periphery [1,36,37]. It is not clear whether CD4^+^CD25^−^Foxp3^+^ cells develop from a distinct precursor than their CD4^+^CD25^+^Foxp3^−^ counterparts or whether this population represents unstable tTregs [38]. Nevertheless, since not all Foxp3^+^ tTregs express CD25, is possible that Foxp3 expression may proceed through an IL-2/STAT5-independent pathway [39]. Thus, the exact mechanism involved in the generation of thymic CD4^+^CD25^−^Foxp3^+^ remains to be determined. Intriguingly, the proportion of Foxp3^+^ cells expressing CD25 increased as a result of infection, mainly after 21 days of infection, coinciding with the minimal IL-2 contents. Interestingly, Tai and colleagues proposed that Foxp3 expression in thymocytes induced pro-apoptotic signals resulting in cell death, provided there is no counterbalance of signals triggered by γc-mediated cytokine receptors [40]. In the context of *T*. *cruzi* infection, CD4^+^Foxp3^+^CD25^−^ cells might be those most prone to Foxp3 induced apoptosis since they do not require γc-mediated signals to survive and complete their differentiation into mature tTreg cells, resulting in a raised proportion of CD4^+^Foxp3^+^CD25^+^ cells. Moreover, the increase of CD25 membrane expression seen after infection may imply that cells with higher CD25 levels, in an environment poor in cytokines critical for their differentiation, can keep a relative efficiency for the IL-2- or IL-15-dependent signaling pathways [6,31,41]. Furthermore, the increased GITR expression may favor anti-apoptotic pathways in CD4^+^CD25^+^Foxp3^+^ cells, reinforcing results about the increased Ki-67/Annexin V ratio [42,43]. Another plausible explanation is that local or environmental inflammatory signals may influence the phenotype of tTregs, as seen in the periphery with T effector and extrathymic DP cells during murine *T*. *cruzi* infection [44].

Confocal microscopy studies performed in normal thymuses showed that Foxp3^+^ cells are located predominantly in the medullary region, in light with the studies by Sakaguchi and Fontenot [45,46]. In the case of infected animals, although positive immunostaining was concentrated mainly on the medulla, the cortex revealed an increase in the presence of Foxp3^+^ cells. This abnormal localization may affect the type of antigens recognized by the tTregs and their selection process, taking into account that medullary and cortical machinery of antigen presentation differs considerably [47]. Undoubtedly, this point deserves to be studied more deeply considering the suspected autoimmune basis of Chagas disease. Besides this, cortical localization of tTregs strongly suggests that suffers intrathymic migratory abnormalities. The fact that infected mice present decreased proportions of α subunits of fibronectin receptors (and also for VCAM-1 in the case of VLA-4) together with the diminished *ex vivo* fibronectin-driven migration of Foxp3^+^ cells point out that tTreg cells may have an abnormal decrease of intrathymic migratory activity, in opposition to other thymic subpopulations that exhibited increased migratory response during *T*. *cruzi* infection [2]. Abnormalities in the expression of migratory-related molecules may affect maturation, stability and selection process of thymocytes [19,48,49], including tTregs, but also could influence the migration of pTregs to target organs during infectious process [50]. The intensity of CD62L expression is closely related to the maturational stage of thymocytes, with Foxp3^+^CD62L^-^ or Foxp3^+^CD62L^low^ cells representing a more immature lineage, whereas Foxp3^+^CD62L^+^ or CD62L^high^ cells being considered more advanced in their development [3]. Increased CD62L expression in Tregs indicates that the intrathymic migration process is modulated by the infection, accelerating the maturation development or favoring their suppressive capacity [51].

A further aspect to be discussed is that a proportion of Foxp3^+^ cells with a marked maturational profile may correspond to peripheral cells that have re-entered the thymus. Under physiological conditions, the re-entry of mature T cells is restricted to activated or memory cells [3,52]. Because there is no marker accurately distinguishing tTregs from pTregs [6], we cannot establish in which proportion these cells come from periphery. However, if this is the case, peripherally activated Foxp3^+^ cells should rather correspond to an effector/memory profile with CD62L^low^ or CD62L^−^ expression, even though central memory populations express CD62L^high^ [48]. Furthermore, we recently reported that during infection, pTregs exhibited a weak capacity to turn into an effector/memory phenotype, as they remain mainly in a naïve state loosing Foxp3 expression. This may be at the origin of impaired pTreg cell function [12], suggesting that the number of pTregs with an activated profile entering the thymus is very low. In any case, the physiological relevance of the re-entry into the thymus of Treg cells is still debatable. Recent data suggest that the re-entry of activated pTregs cells (expressing CD62L^−^CXCR4^+^) into the thymus may inhibit IL-2-dependent differentiation of tTreg cells [47,51]. On the other hand, some authors speculate that the re-entry of T effector cells may contribute to the induction of tolerance by promoting the tTreg development by acting as a source of IL-2 [53]. Considering that the thymus can be infected by *T*. *cruzi* [26], pTregs and T effector cells within the gland may also regulate tTreg differentiation. Whatever the case, remaining tTregs observed during *T*. *cruzi* infection increased and diminished the respective levels of CD62L and CD184/CXCR4 expression, weakening the possibility that the proportional tTreg increase may respond to an enhanced return of activated pTregs.

There is now accumulating evidence in favor that normal thymus continuously produces Treg cells recognizing a broad repertoire of self- and non-self antigens [52]. The clear-cut in trathymic numerical loss of tTregs during the acute phase of *T*. *cruzi* infection may compromise the composition and replenishment of the peripheral tTreg cell pool. If sustained over time, this may have implications in the dysregulated immune responses seen during the chronic phase of this disease. Our previous results also showed that acute infection also induces the collapse of pTregs due to the acquisition of an abnormal Th1-like phenotype and altered functional features [12], reinforcing the idea that *T*. *cruzi* disrupts the Treg response via multiple pathways including central and peripheral mechanisms. This hypothesis is further strengthened by the fact that individuals in the asymptomatic phase of human Chagas disease exhibit a higher proportion of Tregs compared to those who have developed an overt cardiac pathology [7,8].

Our work provides a clear demonstration that *T*. *cruzi* infection impact upon the tTreg cell compartment inducing a noticeable loss of tTreg cells, while the remaining population of tTregs showed an abnormal localization, phenotypic and functional changes. These data, together with the alterations previously described in the peripheral compartment of Tregs [12], suggest that tTreg abnormalities may have harmful consequences for the immunocompetence of the host, further influencing the development of chronic disease. In essence our results provide a stimulating background for further elucidation of important aspects of thymic function in the context of *T*. *cruzi* infection. Future studies performed both in chronic experimental models and humans with Chagas disease will clarify whether tTregs dysfunction may be involved in the suspected autoimmune component of Chagas disease.

## Supporting Information

S1 Fig tTregs dynamics in BALB/c and C57BL/6 mice infected with different strains of *T*. *cruzi*. BALB/c and C57BL/6 male mice were parallel infected with either the Tulahuen or Y strains of *T*. *cruzi*. Samples were obtained at different days post-infection: a) the frequency and b) the absolute number of CD4^+^Foxp3^+^ cells. Values are mean ± s.e.m. of three-six mice/day/group. Representative data from three-five experiments performed independently for each parasite/mice strain pair. * p <0.05 and **p<0.01 as compared to day 0 post-infection.
(TIF)

S2 Fig tTregs frequency during acute and chronic phase of *T*. *cruzi* infection. Using a BALB/c model of infection (100 trypomastigotes of Tulahuén strain inoculated by subcutaneous route) tTregs frequency was monitored at 17 and 21 days p.i. (acute phase) and after 100 days p.i. (chronic phase). Healthy age-matched animals were used as control group. Values are mean ± s.e.m. of three-six mice/day/group. * p <0.05 and **p<0.01.
(TIFF)

S3 Fig Co-expression of typical markers among Foxp3 expressing cells during *T*. *cruzi* infection. Frequency of CD25/GITR and CD25/CD62L co-expression among CD4^+^Foxp3^+^ cells after 21 days p.i. Values are mean ± s.e.m. of 3–8 mice/day. One representative of three experiments performed independently in C57BL/6 mice infected with Tulahuén strain. * p <0.05; and ** p<0.01.
(TIFF)

S4 Fig Frequency of DP within Foxp3 expressing cells. Representative histogram of whole thymocytes expressing Foxp3 (Total Foxp3^+^) plus dot plot representing Foxp3^+^ expressing thymocytes within the different subpopulations (left panel). Bars represent the variation in the proportions of DP thymic Foxp3^+^ expressing cells in control and infected thymus after 21 day p.i (right panel). Data correspond to mean ± s.e.m. of 5 mice/group (one representative round of four independent sets of experiments). * p<0.05.
(TIFF)
