# Supplementary figures and images for: Trypanosoma cruzi Experimental Infection Impacts on the Thymic Regulatory T Cell Compartment
Source: PLoS Negl Trop Dis. 2016 Jan 8;10(1):e0004285. doi: 10.1371/journal.pntd.0004285 (PMC4706328; doi:10.1371/journal.pntd.0004285)

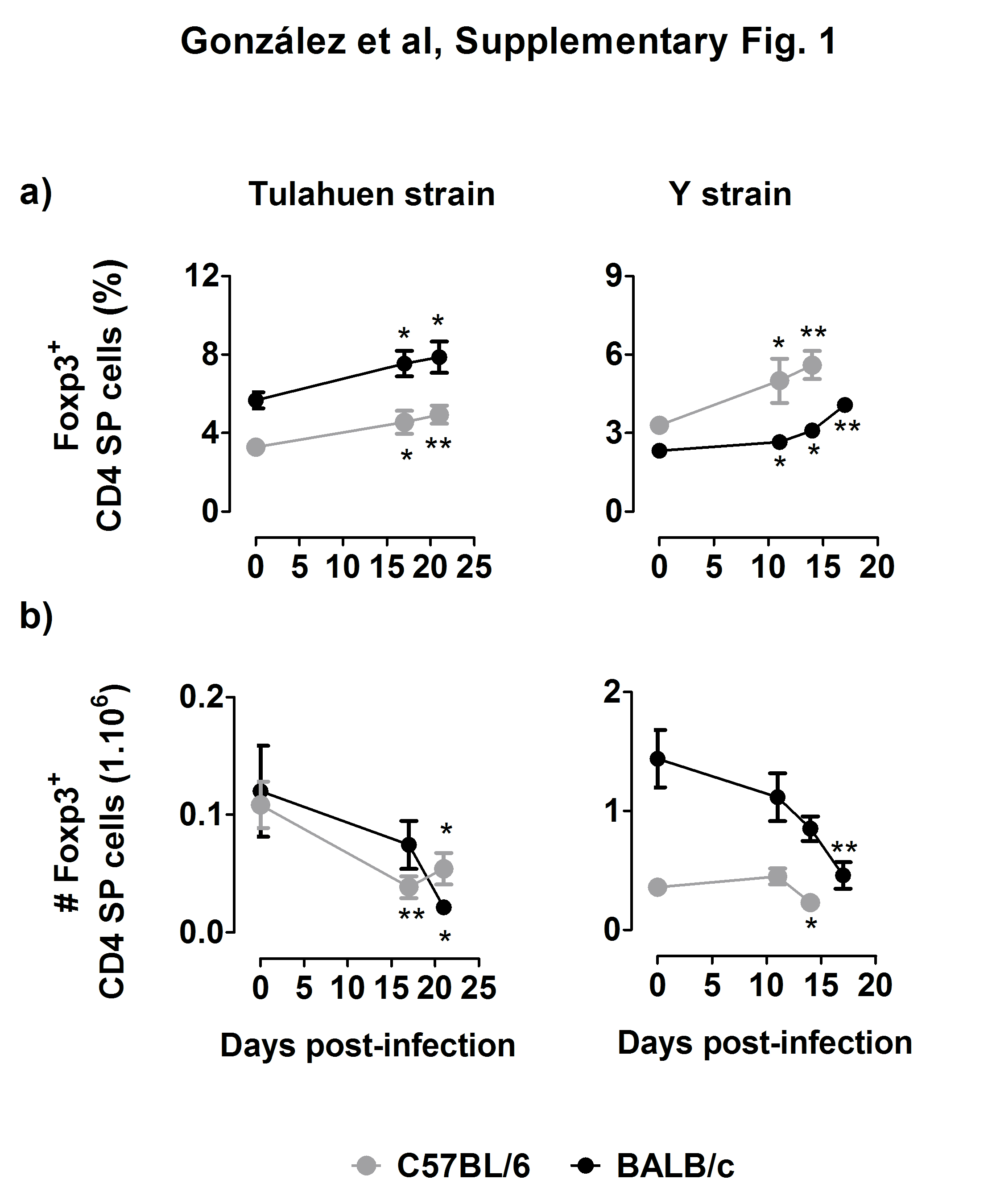

Supplement: S1 Fig — BALB/c and C57BL/6 male mice were parallel infected with either the Tulahuen or Y strains of T. cruzi. Samples were obtained at different days post-infection: a) the frequency and b) the absolute number of CD4+Foxp3+ cells. Values are mean ± s.e.m. of three-six mice/day/group. Representative data from three-five experiments performed independently for each parasite/mice strain pair. * p <0.05 and **p<0.01 as compared to day 0 post-infection. (TIF) [file pntd.0004285.s001.tif]

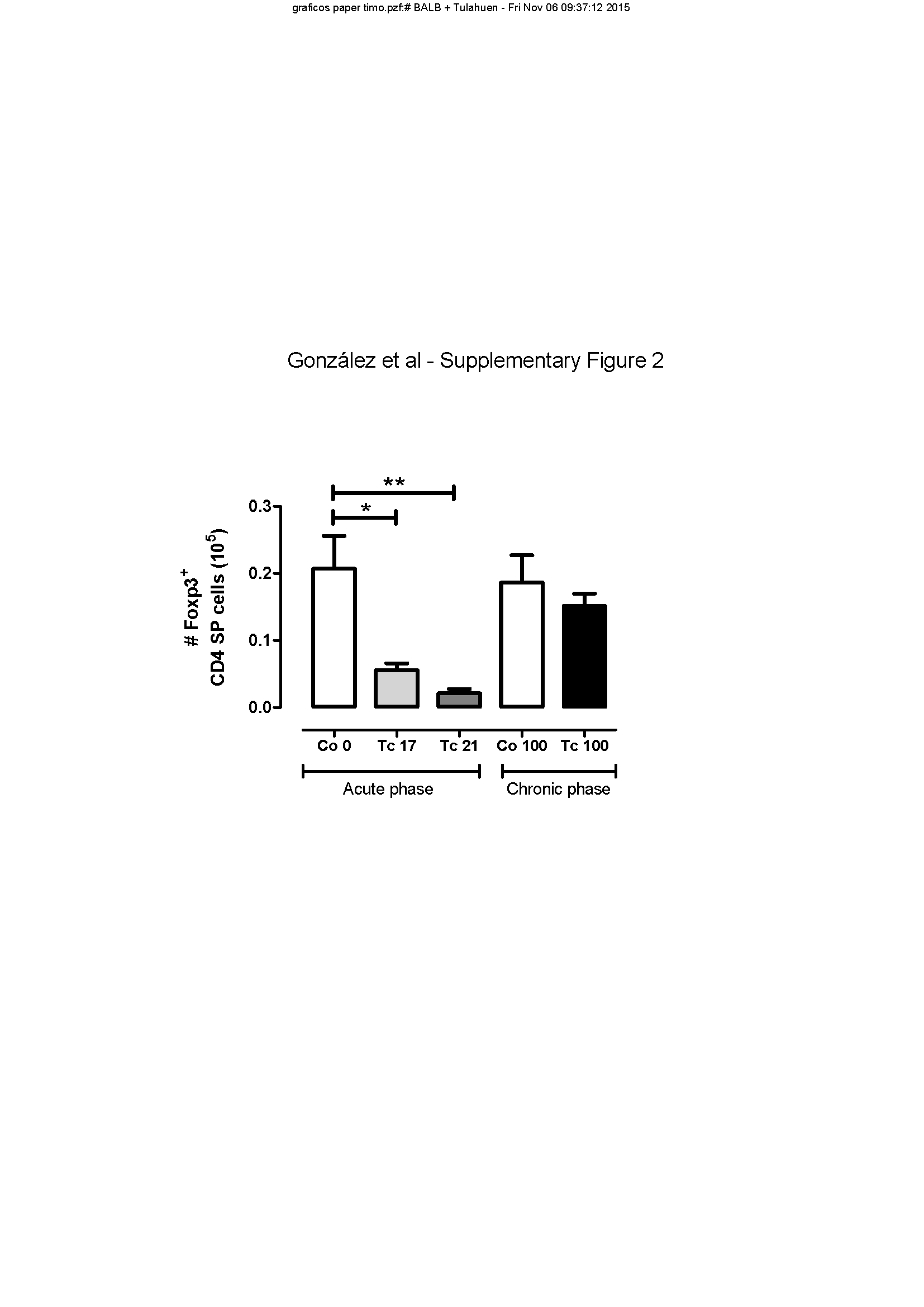

Supplement: S2 Fig — Using a BALB/c model of infection (100 trypomastigotes of Tulahuén strain inoculated by subcutaneous route) tTregs frequency was monitored at 17 and 21 days p.i. (acute phase) and after 100 days p.i. (chronic phase). Healthy age-matched animals were used as control group. Values are mean ± s.e.m. of three-six mice/day/group. * p <0.05 and **p<0.01. (TIFF) [file pntd.0004285.s002.tiff]

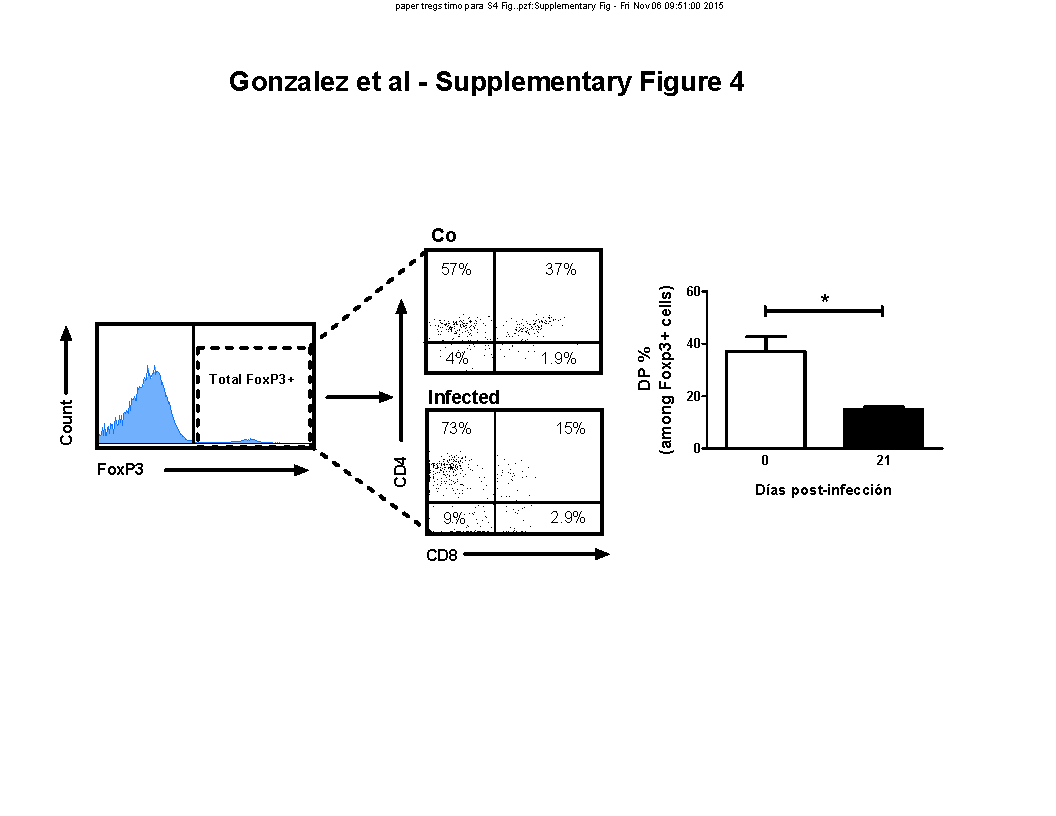

Supplement: S4 Fig — Representative histogram of whole thymocytes expressing Foxp3 (Total Foxp3+) plus dot plot representing Foxp3+ expressing thymocytes within the different subpopulations (left panel). Bars represent the variation in the proportions of DP thymic Foxp3+ expressing cells in control and infected thymus after 21 day p.i (right panel). Data correspond to mean ± s.e.m. of 5 mice/group (one representative round of four independent sets of experiments). * p<0.05. (TIFF) [file pntd.0004285.s004.tiff]
